# Histopathologic diagnosis of chronic graft-*versus*-host disease of the oral mucosa according to the National Institutes of Health Consensus

**DOI:** 10.1590/S1679-45082014AO2974

**Published:** 2014

**Authors:** Paulo Sérgio da Silva Santos, Fábio Luiz Coracin, José Carlos de Almeida Barros, Marina Helena Cury Gallottini

**Affiliations:** 1 Faculdade de Odontologia de Bauru, Universidade de São Paulo, Bauru, SP, Brazil.; 2 Hospital das Clínicas, Faculdade de Medicina, Universidade de São Paulo, São Paulo, SP, Brazil.; 3 Irmandade da Santa Casa de Misericórdia de São Paulo, São Paulo, SP, Brazil.; 4 Faculdade de Odontologia, Universidade de São Paulo, São Paulo, SP, Brazil.

**Keywords:** Graft vs host disease/pathology, Salivary glands/pathology, Hematopoietic stem cell transplantation, Mouth mucosa/pathology

## Abstract

**Objective:**

To validate the minimal criteria of the histopathologic diagnosis of oral chronic graft-*versus*-host disease, based on the histopathologic classification of the National Institutes of Health and correlate them with clinical features.

**Methods:**

Forty-one specimens containing both oral mucosa and salivary glands were analyzed in slides stained with hematoxylin-eosin. The histological specimens were blindly examined by two trained pathologists using criteria recommended for the histopathologic diagnosis of chronic graft-*versus*-host disease proposed by the National Institutes of Health Consensus. The clinical classification of chronic graft-*versus*-host disease was correlated with analysis of slides.

**Results:**

Our data showed that the epithelium was involved in 39/41 specimens, presenting acanthosis (29/70.7%), exocytosis of lymphocytes (29/70.7%), thickening of basal lamina (29/70.7%), and apoptosis (15/36.6%). Connective tissue presented interstitial inflammatory infiltrate (38/92.7%). Minor salivary glands showed periductal fibrosis (38/92.7%), mixed periductal inflammatory infiltrate (32/78%), ductal ectasia (30/73.2%), lymphocytes around and into acinar units (30/73.2%), and interstitial fibrosis (29/70.7%). The most common clinical manifestations were lichenoid aspect (40/97.6%), complaints of sensitivity to oral feeding (38/92.7%), and dry mouth sensation (36/87.8%).

**Conclusion:**

This study validated the National Institutes of Health Consensus of minimal histologic criteria for diagnosis of oral chronic graft-*versus*-host disease and has not found an association between the severity of clinical manifestation and the histopathological stage.

## RESUMO

**Objetivo:**

Validar os critérios mínimos de diagnóstico histopatológico da doença do enxerto contra hospedeiro crônica oral, com base em critérios de classificação do *National Institutes of Health*, e correlacionálos com as características clínicas.

**Métodos:**

Quarenta e um espécimes contendo mucosa oral e glândulas salivares foram analisados em lâminas coradas por hematoxilina-eosina. Os espécimes histológicos foram avaliados de forma cega, por dois patologistas calibrados, utilizando os critérios recomendados para diagnóstico histopatológico de doença do enxerto contra hospedeiro crônica propostos pelo Consenso do *National Institutes of Health*. A classificação clínica da doença do enxerto contra hospedeiro crônica foi correlacionada após a análise das lâminas.

**Resultados:**

Nossos resultados mostraram que o epitélio estava comprometido em 39/41 espécimes, apresentando acantose (29/70,7%), exocitose de linfócitos (29/70,7%), espessamento da lâmina basal (29/70,7%) e apoptose (15/36,6%). O tecido conjuntivo apresentou infiltrado inflamatório intersticial em 38 (92,7%) casos. Nas glândulas salivares menores, observaram-se fibrose periductal (38/92,7%), infiltrado inflamatório periductal misto (32/78%), ectasia ductal (30/73,2%), linfócitos em torno e migrando para dentro dos ácinos (30/73,2%), e fibrose intersticial (29/70,7%). As manifestações clínicas mais comuns foram mucosa de aspecto liquenoide (40/97,6%), queixa de sensibilidade bucal ao se alimentar (38/92,7%), e sensação de boca seca (36/87,8%).

**Conclusão:**

Os critérios mínimos para o diagnóstico histopatológico da doença do enxerto contra hospedeiro crônica oral, com base no Consenso do *National Institutes of Health*, foram validados neste estudo, e não houve correlação entre a gravidade das manifestações clínicas e a gravidade das características histopatológicas.

## INTRODUCTION

Chronic graft-*versus*-host disease (cGVHD) is the major cause of morbidity and mortality in patients undergoing allogenic hematopoietic stem cell transplantation (HSCT), affecting 30 to 50% of transplants^(1)^ and 60 to 80% of long-term survivors^(2)^ involving oral mucosa, skin, liver, gastrointestinal tract, and lymphoid system.^(3)^ Oral mucosa can be the first site affected and its involvement may be seen in up to 80% of patients affected by cGVHD.^(1)^

Main clinical features of oral cGVHD include lichenoid changes, ulcerations and mucosal atrophy, salivary gland dysfunction, superficial mucoceles, reduced mouth opening due to sclerodermatous changes; and, consequently, perioral fibrosis.^(4)^ Buccal mucosa and the lateral and ventral aspects of the tongue are commonly affected. The most common symptoms comprise pain, sensitivity to foods, dry mouth, and alteration in the sense of taste.^(3,5)^ The differential diagnosis can be done among viral infection, drug toxicity, squamous cell carcinoma;^(4)^ and, in those cases, histopathological examination is mandatory. Once detected in the mouth, cGVHD should be investigated in other organs.^(3,6)^

Histological features of oral mucosa cGVHD are not pathognomonic, and the changes affect the epithelium and connective tissue as well as minor salivary glands.^(7,8)^ Beginning with the initial publications of the histopathology of progressive cGVHD, many practical and unsolved issues in the surgical pathology of GVHD are not addressed in standard texts.^(8)^

Horn et al., in 1995, suggested a histological grading of oral cGVHD ranging from grades I to IV according to the alterations in the oral mucosa and salivary glands^(7)^ (Chart 1). Later, in 2006, Shulman et al. discussed the histopathological changes of cGVHD in several organs and suggested a new histopathological classification for this disease named according to the National Institutes of Health (NIH) Consensus' classification^(8)^ (Chart 2). Four diagnostic categories were established by this classification: “without cGVHD,” “possible cGVHD,” “consistent with cGVHD,” and “definite cGVHD”^(8)^ in association with cGVHD clinical features.

**Chart 1:** Horn's chronic graft-*versus*-host diseasehistologic classification of oral mucosa and salivary glands^(7)^

Grade I	Mucosa: vacuolization of basal cells, moderate lymphocytic infiltrate, moderate epithelial exocytosis
	Salivary glands: mild interstitial inflammation
Grade II	Mucosa: epithelial cells with basal vacuolization and dyskeratotic, necrotic keratinocytes with satellitosis, moderate to heavy lymphocytic infiltrate in the submucosa and moderate epithelial exocytosis
	Salivary glands: mild acinar destruction, ductal dilation, squamous metaplasia, mucous pooling, mild fibrosis, duct cell proliferation, periductal lymphocytic infiltrate
Grade III	Mucosa: focal cleavage between the epithelium and connective tissue, intense lymphocytic infiltrate in the connective tissue, dyskeratotic epithelial cells, lymphocyte exocytosis
	Salivary glands: marked interstitial lymphocytic infiltrate. Diffuse destruction of ducts and acini
Grade IV	Mucosa: separation of epithelium and the connective tissue
	Salivary glands: nearly complete loss of acini, dilated ducts, interstitial fibrosis with or without inflammation

**Chart 2:** Shulman chronic graft-*versus*-host diseasehistologic classification of oral mucosa and salivary glands, according to National Institutes of Health Consensus^(8)^

Epithelium	Epithelial thickness (normal, atrophic, hyperkeratosis and acanthosis), presence of vacuolization, apoptosis, spongiosis, atypical keratinocytes, exocytosis of lymphocytes, presence of other inflammatory cells and thickening of basal lamina
Lamina propria	Predominant cell type in the inflammatory infiltrate and their distribution in relation to the salivary duct and epithelium
Salivary glands	Lymphocytes within the duct, periductal mixed infiltrate, presence of lymphocytes within the acini, apoptosis in the ducts and acini, periductal fibrosis, acinar cell degeneration, interstitial fibrosis, duct ectasia and loss of polarity of epithelial cells of the duct

Whereas Horn's classification is a histological grading based on the degree of lymphocytic infiltration and destruction of glandular acini,^(7)^ the NIH Consensus' classification reflects the concept about the presence or absence of cGVHD by highlighting other histological features.

Based on the literature, the consensus work group stated that the minimal histologic criteria for oral cGVHD include localized or generalized epithelial changes, such as lichenoid inflammation, exocytosis, and apoptosis, or the presence of intralobular, periductal lymphocytes with or without plasma cells and exocytosis of lymphocytes into intralobular ducts and acini. Periductal fibrosis (not generalized interstitial fibrosis) is often present.^(8)^ Consensus, also, established the criteria for carrying out the biopsy samples and obtaining specimens of lesions suspected of cGVHD, recommending that the oral mucosa biopsies include epithelium and five lobules, at minimum, of minor salivary glands.^(8)^ It was suggested as well that a standardized form be used by oral pathologists for the inclusion of histological features while carrying out the histopathological examination of such a biopsy.^(8)^ Also, the assessment of cGVHD activity should focus on lobules that are not completely fibrotic to define cGVHD activity, as well as epithelium changes, leading clinicians and pathologists to be aware that premalignant dysplasias and oral cancers often present themselves with a lichenoid appearance.^(8)^

## OBJECTIVE

According to our knowledge, the histopathological do National Institutes of Health Consensus on oral chronic graft-*versus*-host diseasewas not completely validated, and few have commented upon it in the literature thus far. For this reason, the aim of this study was to validate the minimal criteria of the histopathological diagnosis of oral cGVHD and correlate them with clinical features.

## METHODS

Fifty-nine biopsies of oral mucosa previously diagnosed clinically as cGVHD were reviewed. According to the NIH criteria, specimens should be taken from non-ulcerated site and should include at least five underlying salivary gland lobules. For this reason, 10 cases were excluded because there was no epithelium tissue in the paraffin tissue block and also 8 cases for having no salivary gland tissue in the paraffin tissue block, resulting in 41 specimens containing both epithelium and at least 5 lobules salivary glands.

This research was conducted in the hospital *Irmandade da Santa Casa de Misericórdia de São Paulo* and oral pathology laboratory from the *Faculdade de Odontologia da Universidade de São Paulo*, during the period 2008 to 2009. The project was approved by the Research Ethics Committee (FR 170388, protocol 23/2008).

Serial 5*μ*m thick tissue sections were fixed in formalin, paraffin-embedded and stained with hematoxylin-eosin. Two board certified oral pathologists independently performed the microscopic analysis, under light microscope, using the criteria recommended for the histopathological diagnosis of cGVHD proposed by the NIH Consensus and using Horn's classification. In case of disagreement, the slides were reviewed by the same two pathologists in multi-head microscope and the highest degree of histopathological involvement was chosen. The instructions for establishing NIH and Horn classifications were displayed in chart 1 and 2.

The patients had clinical diagnosis of cGVHD established at the moment of the biopsy. Microscopic analyses were performed without knowledge of the clinical classification to reduce bias to define minimal criteria. The two pathologists observed the same slides and reached a consensus diagnosis, twice: once, according to Horn (grade I to IV), and a second time according to the NIH Consensus (without cGVHD, possible cGVHD, Consistent with cGVHD, and definite cGVHD).

At the end of the morphological analyses, each sample was classified in grade I, II, II or IV (according to Horn), and in: “without cGVHD”, “possible cGVHD”, “consistent with cGVHD”, and “definite cGVHD”, according to the NIH Consensus.

The collection of clinical data from medical records was retrospective for each patient. All patients enrolled in the study underwent HSCT, and oral health information was inserted in the charts. Information compiled included demographic data, the underlying disease, time elapsed between transplantation, and oral mucosa biopsy to clinical diagnosis of cGHVD. The cases were clinically classified according to Akpek, from grade zero to 3, considering the presence or absence of lichenoid reactions, desquamative gingivitis, ulcers, pseudomembranes, salivary dysfunction, sensitivity to foods, oral pain, odynophagia, use of analgesics, or the need for enteral feeding. During the clinical data collection, the NIH clinical consensus had not yet been published, therefore Akpek classification was applied.^(9)^

All data were transferred to a form that was specially developed for this study and entered into an Excel spreadsheet for further statistical analysis. The statistical analysis was carried out using Statistical Package for Social Sciences (SPSS), version 16,0, and the researchers assessed the correlation between the histological and clinical classifications. Qualitative variables were described through their frequency, and the continuous variables were described through their mean, standard deviation, standard error of mean, median, minimum, and maximum values. To the descriptive analysis of histological features of oral mucosa, Kruskal-Wallis and Mann-Whitney tests were used.

## RESULTS

The 41 biopsy diagnoses established by the two pathologists, according to the NIH Consensus and Horn's criteria, are described in table 1.

**Table 1:** Histological classification according to the Horn's^(7)^ and consensus's Shulman^(8)^ criteria

Assessment of biopsies according to Horn's and consensus' grading systems		
Horn^(7)^		
	Grade 0	1 (2.5)
	Grade I	4 (9.8)
	Grade II	26 (63.5)
	Grade III	9 (22)
	Grade IV	1 (2.5)
Consensus (Shulman)^(8)^		
	No cGVHD	2 (5)
	Possible cGVHD	16 (39)
	Consistent with cGVHD	15 (36.5)
	Definitive cGVDH	8 (20)

cGVHD: chronic graft-*versus*-host disease.

**Figure 1:**
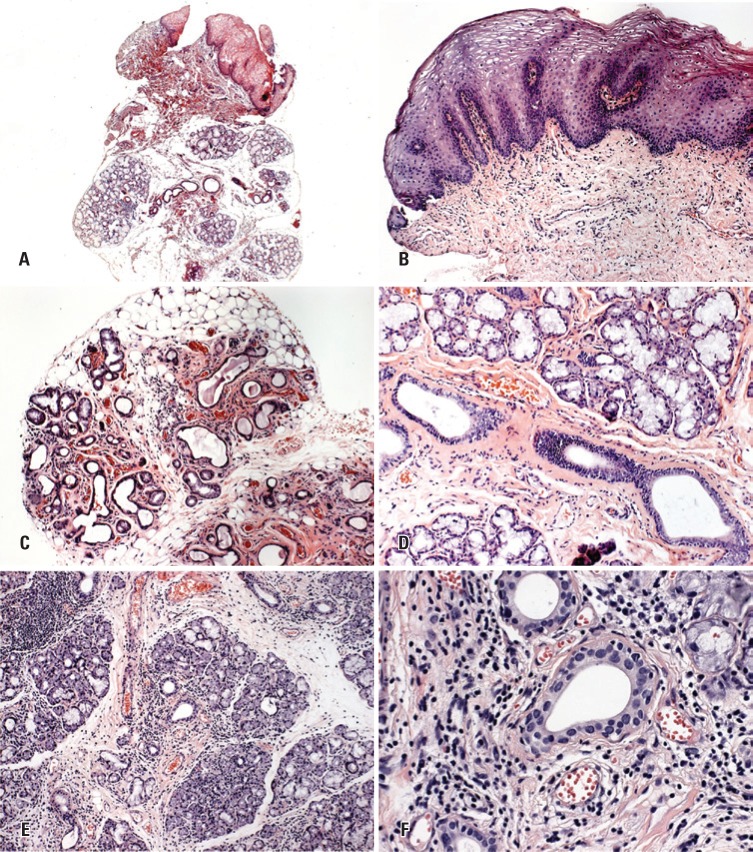
Specimens of oral mucosa biopsy taken from patients with oral chronic graft-*versus*-host disease. (A) Panoramic view of mucosal epithelium and lamina propria with salivary gland lobules. (B) Epithelial hyperplasia and mild inflammatory infiltrate in lamina propria. (C) Ductal ectasia. (D) Periductal fibrosis. (E) Lymphocytes around and migrating into acinar units. (F) High magnification showing periductal mixed chronic infiltrate

Based on the histopathologic criteria for cGVHD diagnosis, the most frequent microscopic alterations observed in the epithelium were: acanthosis in 29 specimens (70.7%); exocytosis of lymphocytes in 29 (70.7%); thickening of the basal lamina in 29 (70.7%); and apoptosis in 15 (36.6%). In the lamina propria, the inflammatory infiltrate was interstitial in 38 (92.7%) cases with lymphocyte predominance in all specimens and, in other cases, plasma cells and eosinophils were found (Figures 1A and 1B). The predominant alterations in the minor salivary glands were: periductal fibrosis in 38 specimens (92.7%), mixed periductal chronic infiltrate in 32 (78%), ductal ectasia in 30 (73.2%), lymphocytes around, and migrating into acinar units in 30 (73.2%); and interstitial fibrosis, in 29 (70.7%) (Figures 1C to 1F).

Generally, in most of the specimens analyzed (36/41; 87.8%), acinar degeneration and/or interstitial fibrosis and/or ductal ectasia were found. This corresponds to an item in the form recommended by the NIH Consensus that includes these three pieces of information (acinar degeneration and/or interstitial fibrosis and/or ductal ectasia). By correlating the final histological diagnosis obtained with the occurrence of each finding belonging to the same item, we found that: regarding acinar degeneration, 8 (32%) were classified as possible cGVHD, 10 (40%) as consistent with cGVHD, and 7 (28%) as definite cGVHD, with no statistically significant difference (p=0.19, Mann-Whitney test); regarding interstitial fibrosis, 8 (27.6%) were classified as possible cGVHD, 13 (44.8%) as consistent with cGVHD, and 8 (27.6%) as definite cGVHD (p=0.01, Mann-Whitney test); and as to ductal ectasia, 12 (40%) were classified as possible cGVHD, 13 (43.3%) as consistent with cGVHD, and 5 (16.7%) as definite cGVHD, with no statistically significant difference (p=0.22, Mann-Whitney test). These data show that in our study interstitial fibrosis played an important role in the differentiation and establishment of the histological grade.

There was an association between NIH Consensus and HORN's criteria histopathological classifications analyzed with Fisher's exact test (p=0.001).

A retrospective clinical analysis of medical records of patients revealed that most lesions that underwent biopsy had lichenoid aspect (40; 97.6%) and that most patients complained of sensitivity to oral feeding (38; 92.7%) and had dry mouth sensation (36; 87.8%). The severity of clinical oral manifestations was not associated with the worse-grading histopathological features in each patient. The clinical graduation of cGVHD was based in Akpek (2001) and we associate with histopathological features (Horn, 1995 and Shulman, 2006) (Table 2).

**Table 2:** Association between clinical grade (Akpek 2001) histological grading (Horn 1995) and consensus (2006)

Patient	Clinical grading (Akpek 2001)	Histological grading (Horn 1995)	Histological grading Consensus NIH (2006)
1	1	III	2
2	1	II	1
3	2	II	2
4	1	III	3
5	2	I	1
6	1	II	1
7	2	II	1
8	2	IV	2
9	1	II	2
10	1	II	3
11	2	II	1
12	1	II	2
13	1	II	2
14	1	II	1
15	1	II	1
16	1	II	2
17	2	II	1
18	1	II	1
19	1	II	1
20	2	II	3
21	1	II	2
22	2	II	3
23	1	II	2
24	1	II	1
25	1	III	3
26	1	III	2
27	1	II	1
28	1	III	2
29	1	I	0
30	2	II	3
31	1	II	1
32	1	I	1
33	1	II	2
34	0	0	0
35	1	II	2
36	1	III	2
37	1	II	2
38	1	I	1
39	1	II	1
40	1	III	3
41	1	III	3

NIH: National Institutes of Health.

## DISCUSSION

Validation studies such as this one are extremely important to define the strengths and weaknesses of the resolutions adopted in the consensus so that it can be improved and modified for better applicability and feasibility. The suggested application of the histopathological criteria of NIH Consensus for oral mucosa and salivary glands may better to characterize the extent of cGHVD.^(10)^ We know that the histopathological findings of oral biopsies after a conditioning regimen are difficult to interpret and that changes after cytotoxic agents can easily be confounded with cGVHD features, mainly those that were performed before the clinical diagnosis of the condition. In this study, all samples were previously diagnosed as cGVHD in oral mucosa and/or salivary glands, and the patients were submitted to the same preparative regimen.

Histological observations of cGVHD lesions are not specific, and the changes may vary, depending on time between HSCT and biopsy, biopsy size, number of serial sections, presence of ulceration area, insufficient depth, and the coexistence of other inflammatory processes at the site.^(11)^ The NIH Consensus histopathological grading system seems more subjective, and for this reason it is preferred by the pathologists. Histopathological criteria for oral cGVHD diagnosis are unspecific compared to other inflammatory conditions due to conditioning regimen. According to the NIH consensus, moderate to intense periductal and periacinar fibrous stroma are evidence of previous inflammation or cGVHD activity, whereas dense fibrous tissue with destruction of acinar tissue and ductal ectasia may be only a marker for previous damage. In this study, salivary gland lobules not completely fibrotic were chosen for histopathological analysis, in an attempt to eliminate the possibility of previous preparative regimen changes/damages from cGVHD changes, as published by Shulman et al.^(8)^ We found periductal and acinar fibrous tissue (92.7%), acinar and periductal inflammation (78%), and damage to ducts comprising ductal lymphocyte exocytosis (73.2%), all of which indicate cGVHD activity.

Histopathological observation of specimens included in this study allows us to infer that grading system proposed by NIH is easily applied, and have an association with Horn's classification. The orientation regarding characteristics of the biopsy and the sequence of observation of microscopic structures facilitate the process of the histological diagnosis.

It was observed that the epithelium of all specimens presented histopathological changes, in particular, acanthosis, exocytosis, apoptosis, thickening of the basal lamina, and keratinocyte atypia. But, unlike the findings of Soares et al.,^(12)^ the occurrence of clefts between the epithelium and connective tissue was rare in our sample. Those authors reported the presence of clefts in 32% of 25 cGVHD cases that were analyzed, whereas we observed only 1 case (2.4%). However, those authors did not describe the clinical aspects of the population studied. The importance of the presence of the cleft between epithelium and lamina propria is questionable, since the consensus recommends performing a biopsy in nonulcerated mucosa areas. In this study, our samples were collected in lichenoid areas but in nonulcerated surfaces.

The minimal histologic criteria for oral cGVHD, such as localized or generalized epithelial changes, lichenoid interface inflammation, exocytosis, and apoptosis or the presence of intralobular, periductal lymphocytes with or without plasma cells and exocytosis of lymphocytes (without neutrophils) into intralobular ducts and acini, are not pathognomonic of GVHD. Apoptosis in the mouth epithelium may not be limited to cGVHD and requires differential diagnosis of cytomegalovirus (CMV) infection.^(8)^ Our main difficulty was the examination of the epithelium at a 10x magnification regarding the quantification of apoptotic cells and the presence of lymphocyte exocytose. In our opinion, the recommended magnification to detect these changes should be at least 40x. Regardless, this recommendation is not completely invalid because its main purpose is to establish the amount of changes per observation field. Even apoptotic bodies are important to the histopathological diagnosis of cGVHD, and we think that these structures can be observed at 40x magnification, as shown by Orti-Raduan et al.^(13)^ Another meaningful observation is that some histopathological alterations, for example acinar apoptosis, could be easily observed in paraffin-embedded tissue cut at a thickness of 3*μ*m instead of 5*μ*m.

The measurement of the thickness of the basal lamina is included in the histopathological guide form, but in a routine stain, it is very difficult to see its width.

Regarding “acinar degeneration/fibrosis/ductal ectasia” of the NIH Consensus form, we observed that there is no balance between these alterations when they are evaluated together, which sometimes made it difficult to establish the final grade. In our analysis, we believe that interstitial fibrosis was the most important criteria for the severity of involvement of the minor salivary glands because 92.7% of salivary glands analyzed showed interstitial fibrosis as the primary manifestation of cGVHD. Therefore, we suggest that this item is broken down into three separate items. These findings are more important regarding cGVHD activity in salivary glands rather than fibrosis in lobules.

Consistent with the findings of other authors,^(14,15)^ we did not find any correlation between clinical (Table 2) and histopathological severity, leading to a nonsynchronous understanding of them. The absence of clinical and histopathological correlation does not diminish the importance of histological analysis of cGVHD, so a differential diagnosis is possible with infectious lesions, drug reactions, or even neoplasias. It is important to establish, upon review of the information on the nonsynchronous clinical and histopathological features of cGVHD, that a correct clinical and histopathological diagnosis needs to be performed. In such cases, the treatments would be totally different, since cGVHD is treated with immunosuppressants.^(16)^

As cGVHD is a multifactorial disease with clinical and histopathologic features in particular, it can often confuse the pathologist. Although salivary glands showed few changes in this study, their analysis must be done carefully because this site can be affected even before the development of mucosal injury. Horn's criteria and the NIH Consensus are different in objective features in the second and in a subjective analysis of nonsynchronous mucosa and salivary glands features in the first.

## CONCLUSION

This study validated the National Institutes of Health Consensus of minimal histologic criteria for diagnosis of oral chronic graft-*versus*-host disease and has not found an association between the severity of clinical manifestation and the histopathological stage.
